# 

**Published:** 1893-07

**Authors:** 


					﻿Vol XIII.
JULY, 1893.
NO. 7?
THE
HOMEOPATHIC pHY^lClAfl,
A Monthly Journal of Homoeopathic Materia Medica
and Clinical Medicine.
•• He it a freeman whom truth makes free, and all are tlavet betide."
“ Seek the Truth : come whence it may, cost what it will."
VDYTKD AND PUBLISHED BY
WALTER N4. JAMES, M. D.
PHILADELPHIA :
No. 1125 8PRUCK 8TREKT.
LOW DO 2<r :
ALFRED HEATH & CO., Sole Agents fob Gbkat Bbitain,
114 Ebury Street, Eaton Square.
<J“FearZj/ SufteertpZion, $2.50, invariably in advance. Single numbers, 25 ets.
Entered at the PbDadelpbia Pwt-Offlee M Seoeod-Claae Hail Hatter.
				

